# Hydroclimatic variability is associated with gastrointestinal parasite dynamics in black-horned capuchins (*Sapajus nigritus cucullatus*) in the Atlantic Forest of Argentina

**DOI:** 10.3389/fvets.2026.1959428

**Published:** 2026-10-02

**Authors:** Gimena Illia, Martin Kowalewski, Felix Ignacio Contreras, Thomas R. Gillespie, Karen E. DeMatteo, Luciana Oklander

**Affiliations:** 1 Estación Biológica Corrientes (EBCo), Centro de Ecología Aplicada del Litoral, Consejo Nacional de Investigaciones Científicas y Técnicas, San Cayetano, Argentina; 2 Laboratorio de Geografía Física, Centro de Ecología Aplicada del Litoral, Consejo Nacional de Investigaciones Científicas y Técnicas, Corrientes, Argentina; 3 Departments of Environmental Sciences and Environmental Health, Rollins School of Public Health, Emory University, Atlanta, GA, United States; 4 Department of Biology, Washington University in St. Louis, St. Louis, MO, United States; 5 WildCare Institute, St. Louis, MO, United States; 6 Neotropical Primate Conservation Argentina, Posadas, Argentina; 7 Instituto de Biología Subtropical, Consejo Nacional de Investigaciones Científicas y Técnicas, Posadas, Argentina

**Keywords:** climatic variables, drought index, monkeys, primate, SPEI, wildlife health

## Abstract

Understanding the environmental drivers of parasite dynamics in wildlife is critical for predicting disease risk under changing climatic conditions. We investigated the relationship between climatic variables and patterns of gastrointestinal parasitism in wild black-horned capuchin monkeys (*Sapajus nigritus cucullatus*) inhabiting the Atlantic Forest (Misiones, Argentina). A total of 131 fecal samples were collected from two study sites (COR: 25°51’S, 53°57’W; YL: 25°34’S, 54°4’W) between May 2019 and November 2021 and analyzed using flotation and sedimentation techniques. Environmental data (temperature, humidity, and precipitation) were obtained from NASA MERRA-2, and the Standardized Precipitation Evapotranspiration Index (SPEI, 3-month scale) was used to characterize moisture availability. Generalized linear mixed models indicated that SPEI was the most informative environmental predictor: both parasite frequency and richness were positively associated with wetter conditions (higher SPEI values), whereas seasonal categories showed no significant effects. At the taxon level, *Strongyloides* sp. occurrence increased with SPEI, while rhabditoid larvae increased with relative humidity and Hymenolepididae decreased with humidity. These findings suggest that continuous variation in moisture availability, rather than discrete seasonal categories, better captures parasite dynamics in this subtropical system. SPEI emerges as a valuable integrative index for capturing environmentally driven variation in parasite communities, particularly under pronounced climatic anomalies such as the 2019–2021 regional drought associated with the onset of La Niña conditions in 2020. Incorporating such indices into wildlife disease ecology may improve the prediction and monitoring of parasite dynamics under ongoing climate change.

## 1 Introduction

Gastrointestinal parasites are ubiquitous components of wildlife communities, and their prevalence and richness can be shaped by a variety of biological and environmental factors (1–4). Although many gastrointestinal helminths and protozoa are obligate endoparasites, they depend on the external environment to complete their life cycles and ensure transmission, as their free-living stages (eggs, cysts, and larvae) develop and persist outside the host (5–8). Because these stages lack the physiological buffering provided by a host, they are particularly sensitive to ambient microhabitat conditions and must maintain viability long enough to reach a new susceptible individual (9). Consequently, climatic conditions such as temperature, humidity, and precipitation play an important role in regulating the distribution, transmission and development of parasites (10–12). Warmer temperatures can accelerate parasite developmental rates, while adequate moisture availability is a key determinant of infective stage survival and parasite pressure in the environment (5, 13–15). In general, higher parasite prevalence and richness have been reported during warmer and wetter periods, when environmental conditions promote the survival of free-living stages and hosts may exhibit behaviors that increase transmission risk (16–19). However, temperature, humidity, and precipitation are often strongly correlated (20–22), making it difficult to disentangle their independent contributions to host–parasite interactions.

Many parasitological studies have relied on broad seasonal categories (e.g., wet vs. dry season, or four calendar seasons) as proxies for environmental variation (16, 18, 19, 23, 24). While this approach can be informative in tropical systems with strongly defined rainfall regimes, it may be less appropriate in subtropical regions such as the Upper Paraná Atlantic Forest (UPAF), where precipitation is irregularly distributed throughout the year and includes variable dry periods (25, 26). In such contexts, discrete seasonal classifications may fail to capture relevant environmental variation, highlighting the value of using continuous or integrative climatic variables instead.

The Standardized Precipitation Evapotranspiration Index (SPEI) is a multiscalar drought index that integrates information on both precipitation and atmospheric evaporative demand (27–29). Unlike single-variable indices, SPEI captures the net water balance of the environment, making it sensitive to both wet and dry periods. At a 3-month temporal scale, SPEI is useful for detecting short- to medium-term changes in moisture availability that correspond to seasonal fluctuations (28, 30). Although SPEI has been widely applied in agricultural and hydrological sciences (27), its use in disease ecology and parasitology is still incipient, with published examples emerging in the last few years (31, 32). Growing evidence suggests that composite drought indices incorporating water balance components may capture environmental conditions relevant to pathogen transmission more effectively than single climatic variables alone (28, 33–35). In the context of infectious diseases, SPEI has been used to investigate drought-driven reemergence of yellow fever virus in southeastern Brazil (36), associations between long-term drought and childhood diarrhea in 51 low- and middle-income countries (37), drought-related cholera outbreaks in sub-Saharan Africa (38), and the effects of drought on health and helminth burden in eastern grey kangaroos in Australia (39). These studies collectively suggest that SPEI could be a promising tool for understanding how moisture availability influences host–parasite interactions. By integrating water balance over biologically relevant time windows, SPEI may better reflect the cumulative moisture conditions that govern the survival, development, and persistence of free-living infective stages in the environment. However, its potential in wildlife parasitology remains largely untested, and to our knowledge, no study has yet explored its applicability to gastrointestinal parasite dynamics in platyrrhine primates.

The Atlantic Forest is one of the most biodiverse and threatened biomes on Earth, recognized as a global biodiversity hotspot with conservation priority (40, 41). Originally spanning approximately 1,700,000 km^2^, more than 92% of its original extent has been lost due to anthropogenic land-use change (42, 43). Among the ecoregions comprising this biome, the UPAF is the most extensive ecoregion of this biome, originally covering ~471,200 km^2^ (25), and today represents a heavily fragmented and climatically heterogeneous subtropical system, well suited for examining environment–parasite interactions. In Argentina, the province of Misiones retains approximately 50% of its original forest cover (44, 45), making it a critical refuge for the biodiversity of the UPAF and an important system for studying environment-parasite interactions in a landscape shaped by both natural climatic variability and anthropogenic disturbance.

The black horned capuchin monkey (*Sapajus nigritus cucullatus*) is a primate whose geographic distribution is restricted to the Atlantic Forest, occurring in southeastern and southern Brazil and in the province of Misiones, Argentina (46). Like other species of the genus *Sapajus*, it is a diurnal, arboreal primate with a generalist and flexible diet, essentially frugivorous-insectivorous, complementing its diet with seeds, leaves, and other plant parts during periods of fruit scarcity (47, 48). This species plays an important ecological role in the forests it inhabits, acting as seed disperser and pollinator (49, 50). Although classified as Near Threatened by the IUCN, in Argentina it is categorized as Vulnerable due to ongoing habitat loss, fragmentation, and additional threats such as road mortality and illegal trade (50, 51). The overlap between the habitat of *S. n. cucullatus* and anthropogenic activities, including agriculture, cattle ranching, and forestry plantations, is well documented (49, 52). Their terrestrial foraging habits and proximity to human-modified environments increase their exposure to a diverse array of gastrointestinal parasites, including taxa shared with domestic animals and humans (53, 54).

In this study, we explored the potential of SPEI as a predictor of gastrointestinal parasite frequency and richness in wild *S. n. cucullatus* inhabiting the UPAF. Additionally, we assessed the relative influence of humidity and seasonal categories on parasite dynamics in a subtropical system, which lacks a strongly defined dry season. We hypothesized that, if SPEI captures biologically meaningful variation in moisture conditions, parasite frequency and richness would increase under wetter conditions (positive SPEI values), reflecting enhanced survival, development, and environmental persistence. We further predicted that SPEI would outperform conventional seasonal categories as a predictor in this system.

## 2 Materials and methods

### 2.1 Study area and sampling

The study was conducted in the UPAF in the department of Manuel Belgrano, Misiones Province, Argentina. The UPAF is in the southern portion of the Atlantic Forest, farthest from the oceanic coast, and has a subtropical climate with pronounced thermal seasonality (mean annual temperature 16–22 °C) and relatively high but irregularly distributed annual precipitation (1,200–2,000 mm) (25). The ecoregion has experienced intense habitat loss and fragmentation driven by urban growth, industrial development, and agricultural expansion (55).

Two study sites were selected, both embedded within the Green Corridor of Misiones, which encompasses approximately 11,000 km^2^ of connected forest remnants (55): (i) the Corredor Biológico Urugua-í–Foerster (COR; 25°51′S, 53°57′W), a landscape mosaic of small farms, private reserves, and protected areas; and (ii) Yacutinga Lodge (YL; 25°34′S, 54°4′W), a 570-ha private reserve dedicated to ecotourism and native forest conservation (Figure 1). The two sites are located less than 30 km apart and share similar climatic conditions. One social group of *S. n. cucullatus* was monitored at each site. The COR group comprised between 12 and 16 individuals ranging over 394 ha, whereas the YL group comprised between 30 and 35 individuals ranging over 241 ha (54). Samples were collected opportunistically while locating and following the groups. Within each field campaign, individuals were provisionally distinguished on the basis of natural physical features (e.g., pelage marks, body size, scars) to avoid re-sampling the same individual on consecutive days; however, reliable individual identification could not be maintained between campaigns.

**Figure 1:**
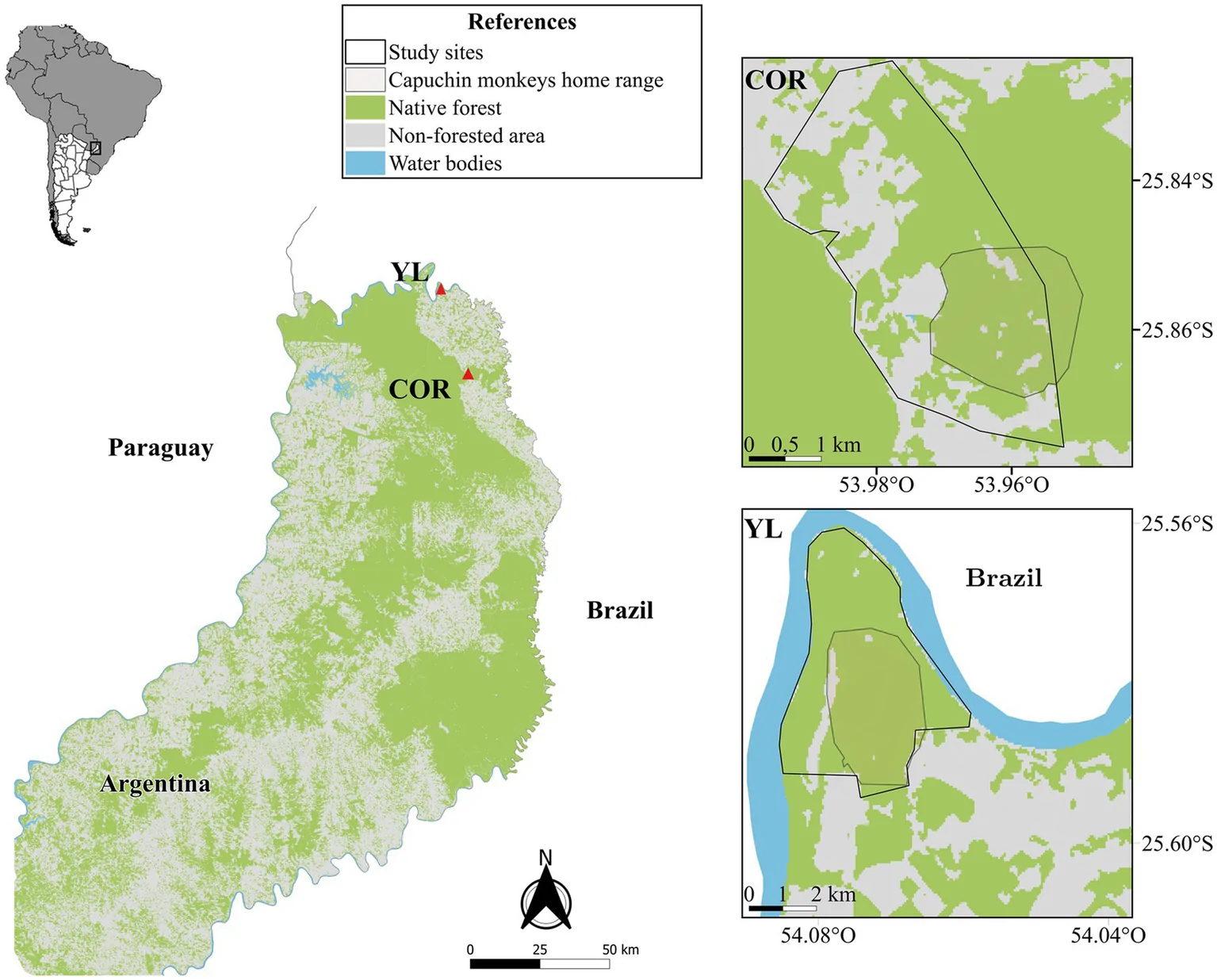
Location of the two study sites in the Atlantic Forest, Misiones Province, northeastern Argentina. COR (Corredor Biológico Urugua-í–Foerster; 25°51′S, 53°57′W) and YL (Yacutinga Lodge; 25°34′S, 54°4′W) are indicated by red triangles. Insets show each site in detail, with the home range of the monitored *Sapajus nigritus cucullatus* group (outlined).

Fecal sampling was carried out from May 2019 to November 2021, across two field phases. Phase I (May 2019–March 2020) consisted of opportunistic collection during direct group follows, conducted over monthly field campaigns of approximately five consecutive days at YL and 7–10 consecutive days at COR, over eight months. The longer campaigns at COR reflected the greater difficulty of locating the group on that site relative to YL. Sampling was interrupted before completion due to COVID-19 restrictions. Phase II (January–November 2021) resumed under institutional biosafety protocols and incorporated a trained detection dog to improve fecal sample recovery. The dog was trained to locate fresh capuchin feces by smell; all candidate samples identified by the dog were subsequently inspected and confirmed by the researchers prior to collection to ensure correct assignment (54). Although sampling procedures differed between phases, fecal samples in both cases were collected exclusively upon direct visual confirmation of the defecating individual. For each sample, date, time, and GPS coordinates were recorded. Samples were preserved in 10% formalin for subsequent parasitological analysis.

All fieldwork was conducted using noninvasive fecal sample collection methods. Sample size was determined by opportunistic encounter rather than by a predetermined target. All collections were authorized by the Ministerio de Ecología y Recursos Naturales Renovables (MEyRNR) of Misiones, Instituto Misionero de Biodiversidad (IMiBio), Administración de Parques Nacionales (APN), and the Instituto Nacional de Tecnología Agropecuaria (INTA) (Resolutions: 008/19, 020/19, 002/20, 003/21). The fieldwork followed biosafety protocols and recommendations established by the Sociedad Argentina para el Estudio de los Mamíferos (SAREM; https://www.sarem.org.ar/acerca-de-la-covid-19/) and the Sociedad Latinoamericana Primatología (SLAPRIM; Comunicado sobre primates, COVID-19 y SARS-CoV-2: https://slaprim.org/avisos/) to minimize pathogen transmission between researchers and wildlife.

### 2.2 Parasitological analysis

Fecal samples were processed using standard coprological techniques: flotation with Sheather’s sugar solution (specific gravity 1.250–1.270) and spontaneous sedimentation (56). Each sample was examined in duplicate under light microscopy. Parasite stages (eggs, cysts, and larvae) were photographed and measured using Leica Application Suite software and identified to the lowest possible taxonomic level using published keys and checklists (53, 57–61).

Two parasitological indices were used as response variables in statistical models: parasite frequency (PF), defined as the proportion of samples testing positive for at least one parasite taxon; and parasite richness (PR), defined as the number of distinct parasite taxa detected per sample. For taxon-specific analyses, the presence or absence of each taxon in each sample was used as a binary response variable. Taxa detected in fewer than five samples across the entire dataset were classified as rare and excluded from individual models to avoid results biased by low occurrence frequencies.

### 2.3 Environmental variables

Climatic data for the study period (2019–2021) were obtained from the NASA MERRA-2 atmospheric reanalysis system (97). Given the proximity of the two sites (Section 2.1), identical environmental data were applied to both. The following variables were extracted or derived: (1) mean temperature (tmed): mean daily air temperature at 2 m height (°C); (2) relative humidity (hmed): mean daily relative humidity at 2 m height (%); (3) precipitation (precip): daily accumulated precipitation (mm); (4) SPEI at a 3-month temporal scale (SPEI-3), which was computed monthly using the SPEI package in R (Begueria and Vicente-Serrano, 2023). Each monthly value summarizes the climatic water balance accumulated over the focal month and the two preceding months, providing a single continuous time series with monthly resolution rather than non-overlapping 3-month blocks. SPEI values ranged from −3 (extreme drought) to +3 (extreme wet conditions) (28). The 3-month scale was selected to capture short- to medium-term moisture variability relevant to seasonal parasite dynamics; season: categorized as summer (January–March), autumn (April–June), winter (July–September), and spring (October–December). (5) Season was included to allow comparison between conventional categorical approaches and continuous climatic indices. All climatic variables were derived at monthly resolution and assigned to samples according to their collection month. Samples within the same calendar month shared identical predictor values. The environmental predictors therefore took 14 values across the study period, corresponding to the 14 sampled month × year combinations.

### 2.4 Statistical analysis

To assess the effects of environmental variables on parasite community dynamics, generalized linear mixed models (GLMMs) were fitted using the complete dataset (*n* = 131 fecal samples). PF and PR were used as response variables in separate models. Season, SPEI, mean temperature, precipitation, and relative humidity were included as fixed effects. Social group (COR and YL) and calendar year were included as crossed random intercepts. No data cleaning or imputation was required prior to modeling, all 131 samples had complete records for the response and predictor variables, and no observations were excluded based on missing data. To control temporal autocorrelation and pseudoreplication across the sampling period, we first controlled the models using calendar month nested within year was included as an additional random effect to control. However, because SPEI and mean relative humidity were computed at monthly resolution, a month-within-year random term varies at the same scale as these fixed predictors and is confounded with them; consistent with this, models specifying month nested within year produced singular fits in which the group and year variances were estimated at zero. Calendar month was therefore not retained as a random effect. Sampling was irregular, with several months without samples; time was therefore represented by the actual dates of collection rather than as a continuous monthly series, and unsampled months were not imputed. A full model incorporating all five environmental predictors was initially fitted. Multicollinearity was assessed using Variance Inflation Factors (VIF), with VIF > 5 taken as the threshold for problematic collinearity, complemented by pairwaise Pearson correlations. Precipitation and mean temperature were subsequently removed from the models due to high collinearity with SPEI and relative humidity, respectively. The reduced candidate models therefore included all combinations of season, SPEI, and relative humidity as fixed effects. Models were fitted by maximum likelihood. Parasite frequency was modeled with a binomial error distribution and logit link, while parasite richness was modeled using a Poisson (or negative binomial, if applicable) distribution.

All candidate models were compared against the corresponding null model (intercept + random effects only) using likelihood ratio tests (LRT) following a chi-squared distribution (62). Candidate models were defined as combinations of season, SPEI, and relative humidity and compared each candidate model with the corresponding null model using likelihood ratio tests. No interaction terms were included because we had no prior hypotheses predicting interactive effects among the climatic predictors. When more than a single model was statistically significant (*p* < 0.05) for the same response variable, the model with the lowest Akaike Information Criterion (AIC) was selected as the best-fitting model. *Post hoc* Tukey tests were applied for pairwise comparisons involving the categorical predictor season.

Taxon-specific analyses were performed by fitting separate GLMMs for each parasite taxon, using binary presence/absence per sample as the response variable, with the same fixed-effect predictors and random-effects structure described above. Rare taxa, as defined above (Section 2.2), were excluded from these analyses.

For all final models residuals were tested for uniformity (Kolmogorov–Smirnov test), overdispersion, and outliers, and were inspected for residual patterns against model predictions; zero-inflation was additionally tested for the parasite richness model, and residual temporal autocorrelation was evaluated using a Durbin–Watson test on residuals aggregated by sampling date.

All statistical analyses were performed in R 4.2.2 (63) using the packages lme4 (64), lmtest (65), DHARMa (66), ggeffects (67), car (68), sjPlot (69) and SPEI (70).

## 3 Results

A total of 131 fecal samples from *Sapajus nigritus cucullatus* were collected at the two study sites, corresponding to two social groups (Supplementary Table S1). At least one parasite taxon was detected in 45.8% (60/131) of samples. Parasite frequency was 43.1% (22/51) at COR and 47.5% (38/80) at YL. Fifteen distinct taxa were identified, encompassing protozoa, nematodes, acanthocephalans, and cestodes. Parasite community composition and structure have already been described (54).

### 3.1 Environmental conditions during the study period

During the study period (2019–2021), mean ambient temperature ranged from 15.1 to 30.9 °C (absolute minimum: −1.0 °C; absolute maximum: 39.7 °C). Mean relative humidity ranged from 47 to 89%, and daily precipitation from 5.3 to 305.9 mm (Figure 2). SPEI at the 3-month scale revealed pronounced hydroclimatic variability throughout the study period (Figure 3). Conditions were predominantly dry, with only three wet periods (SPEI > 0) interspersed among prolonged drought episodes (SPEI < 0). Extreme drought conditions (SPEI < −2.0) were recorded during 2020 and late 2021, coinciding with the La Niña event that began in 2020 (71); however, regional moisture deficits in Misiones were already evident in 2019, before that onset. SPEI values during wet periods did not exceed +2.0, indicating that no flooding events occurred.

**Figure 2:**
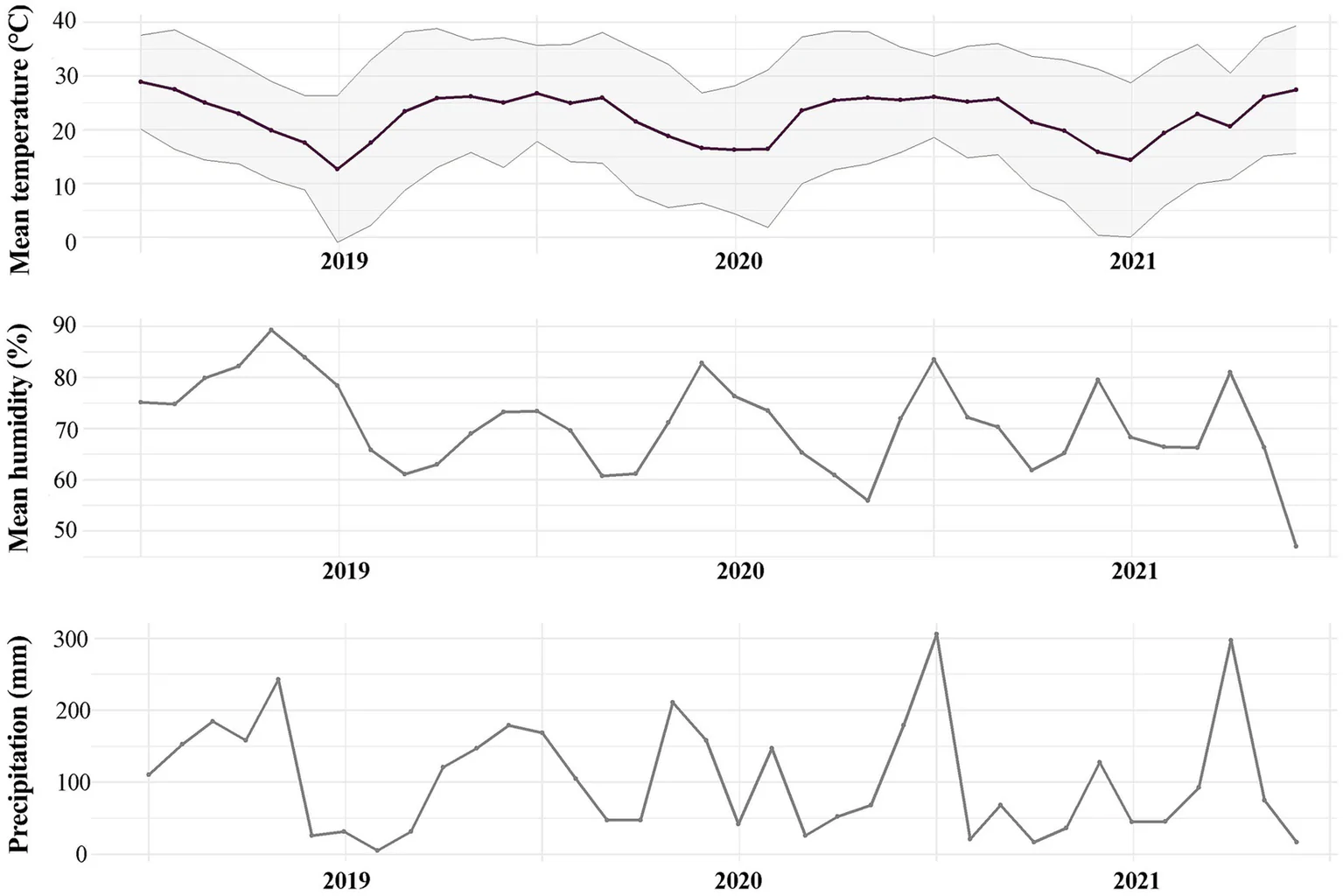
Monthly records of mean daily temperature (°C), mean daily relative humidity (%), and mean daily precipitation (mm) from January 2019 to December 2021 in northeastern Misiones Province, Argentina. Data source: NASA MERRA-2.

**Figure 3:**
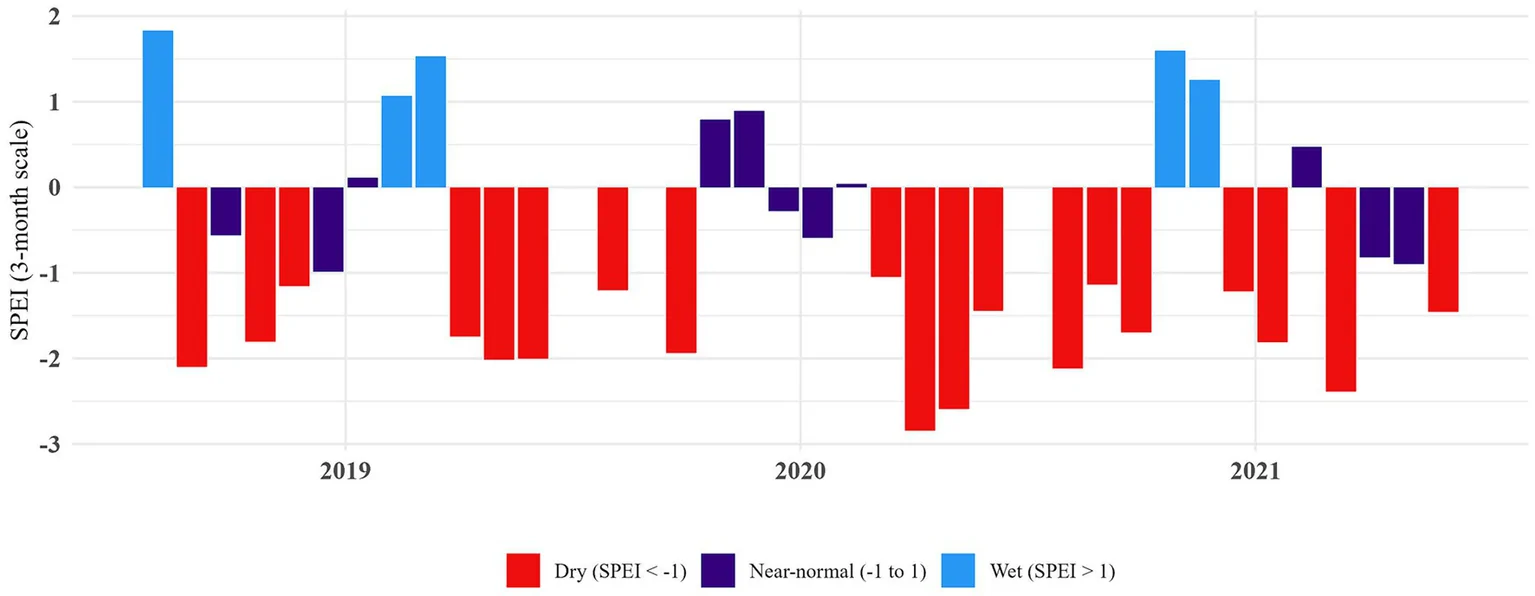
Monthly time series of the Standardized Precipitation Evapotranspiration Index (SPEI) calculated at a 3-month temporal scale from January 2019 to December 2021 for the study area in the northeastern Misiones Province, Argentina. Each bar represents one month. Blue bars indicate wet conditions (SPEI > 1), red bars indicate dry conditions (SPEI < −1), and values between −1 and 1 represent near-normal environmental conditions.

### 3.2 Environmental predictors of parasite community dynamics

Pairwise Pearson correlations among the four candidate environmental variables (SPEI, mean temperature, precipitation, relative humidity) confirmed strong collinearity, particularly between precipitation and mean temperature (*r* = −0.74, *p* < 0.001), precipitation and relative humidity (*r* = 0.74, *p* < 0.001), mean temperature and relative humidity (*r* = −0.56, *p* < 0.001), SPEI and precipitation (*r* = 0.41, *p* < 0.001) and SPEI and mean temperature (*r* = −0.61, *p* < 0.001). In the full model, variance inflation factors approached the threshold for two predictors (VIF: SPEI = 1.62, tmed = 2.90, precip = 3.31, hmed = 2.25). The strength of pairwise associations justified excluding mean temperature and precipitation from subsequent models; precipitation is, in addition, already integrated into the SPEI index. The reduced model (Response – season + SPEI + hmed) showed negligible collinearity (VIF = 1.06 for both SPEI and hmed).

With respect to overall parasite community metrics (parasite frequency and parasite richness), SPEI was the only significant predictor, and season was not significant in any model. Parasite frequency increased significantly with SPEI, indicating that wetter conditions were associated with a higher probability of parasitism (Table 1; Figure 4A). Parasite richness, modeled with a negative binomial distribution, showed the same positive trend with SPEI (Table 1; Figure 4B), although this relationship was comparatively weak and borderline significant (*p* = 0.04). No overdispersion or zero-inflation was detected in the richness model (*p* = 0.94 and *p* = 0.86, respectively).

**Table 1:** Environmental predictors from generalized linear mixed models (GLMMs) for parasite frequency (PF), parasite richness (PR), and parasite taxa in *Sapajus nigritus cucullatus* from January 2019 to December 2021 in northeastern Misiones Province, Argentina (*n* = 131 fecal samples).

Response variable	Predictor	Distribution	*β* (SE)	OR/IRR (95% CI)	*χ* ^2^	AIC	AIC_null_	*p*-value
PF	SPEI	Binomial	0.375 (0.169)	1.46 (1.04–2.03)	5.12	183.56	186.68	0.02
PR	SPEI	Neg. binomial (θ = 1.90)	0.223 (0.112)	1.25 (1.00–1.56)	3.45	324.62	326.07	0.04
Rhabditoid larvae	hmed	Binomial	0.082 (0.036)	1.09 (1.01–1.17)	6.00	114.86	125.72	0.01
Hymenolepididae	hmed	Binomial	−0.058 (0.029)	0.94 (0.89–1.00)	4.23	139.70	141.90	0.04
*Strongyloides* sp.	SPEI	Binomial	0.541 (0.241)	1.72 (1.07–2.76)	4.46	121.98	124.43	0.03

For each response variable, the best-fitting model is shown with its error distribution, coefficient estimate (*β* ± SE), effect size (odds ratio [OR] for binary responses or incidence rate ratio [IRR] for richness) with 95% confidence intervals, likelihood-ratio test statistic (*χ*^2^) against the null model, associated *p*-value, and Akaike Information Criterion (AIC).

**Figure 4:**
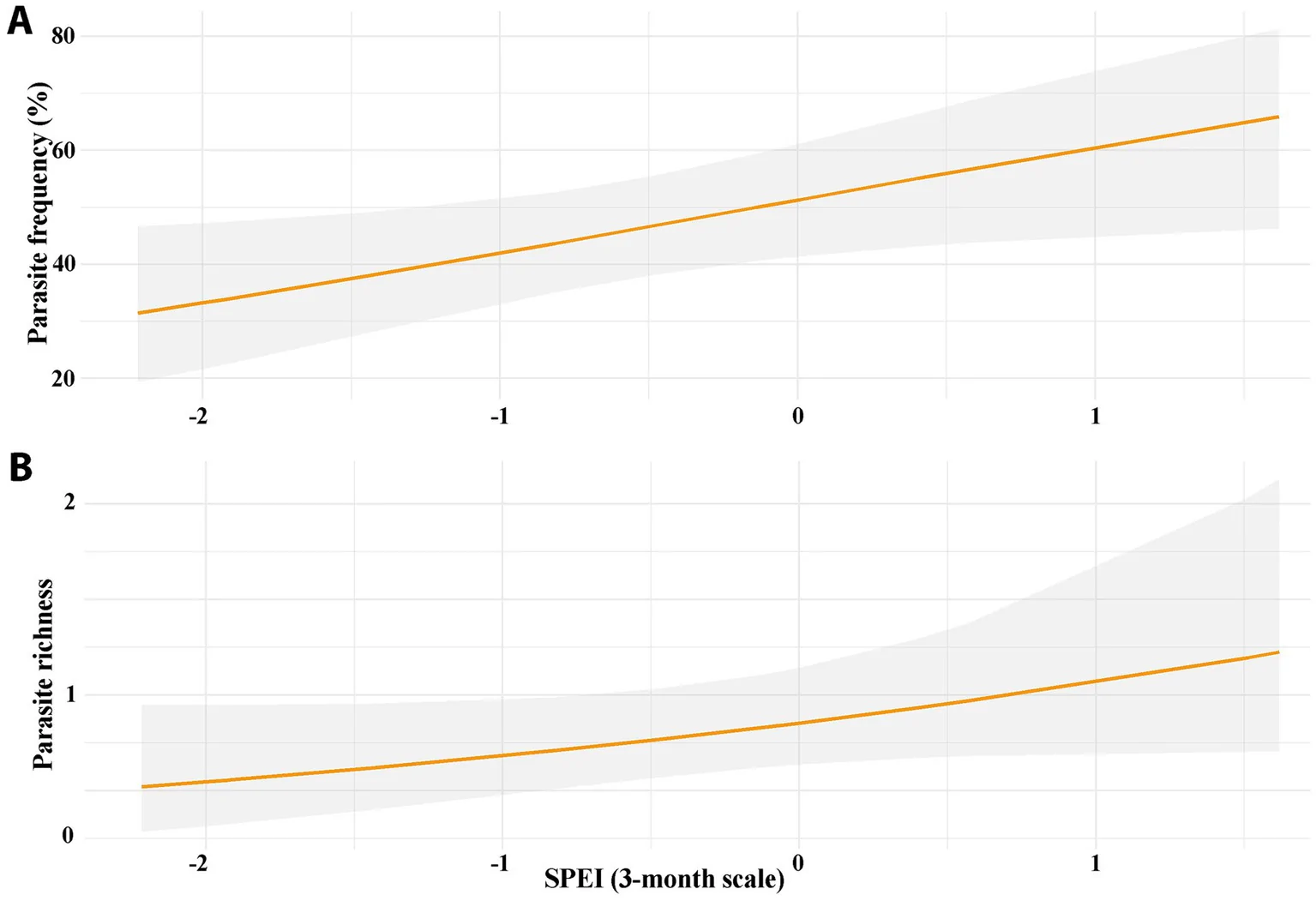
Predicted parasite frequency (%) **(A)** and parasite richness **(B)** in relation to Standardized Precipitation Evapotranspiration Index (SPEI) in *Sapajus nigritus cucullatus* from January 2019 to December 2021 in northeastern Misiones Province, Argentina. Gray shading represents 95% confidence intervals from generalized linear mixed models (GLMMs).

At the taxon level, Rhabditoid larvae occurrence showed a positive association with mean relative humidity (Table 1; Figure 5A), whereas Hymenolepididae occurrence decreased with increasing relative humidity (Table 1; Figure 5B). The occurrence of *Strongyloides* sp. increased with SPEI (Table 1; Figure 5C). Model outputs, including effect sizes and 95% confidence intervals, are presented in Table 1.

**Figure 5:**
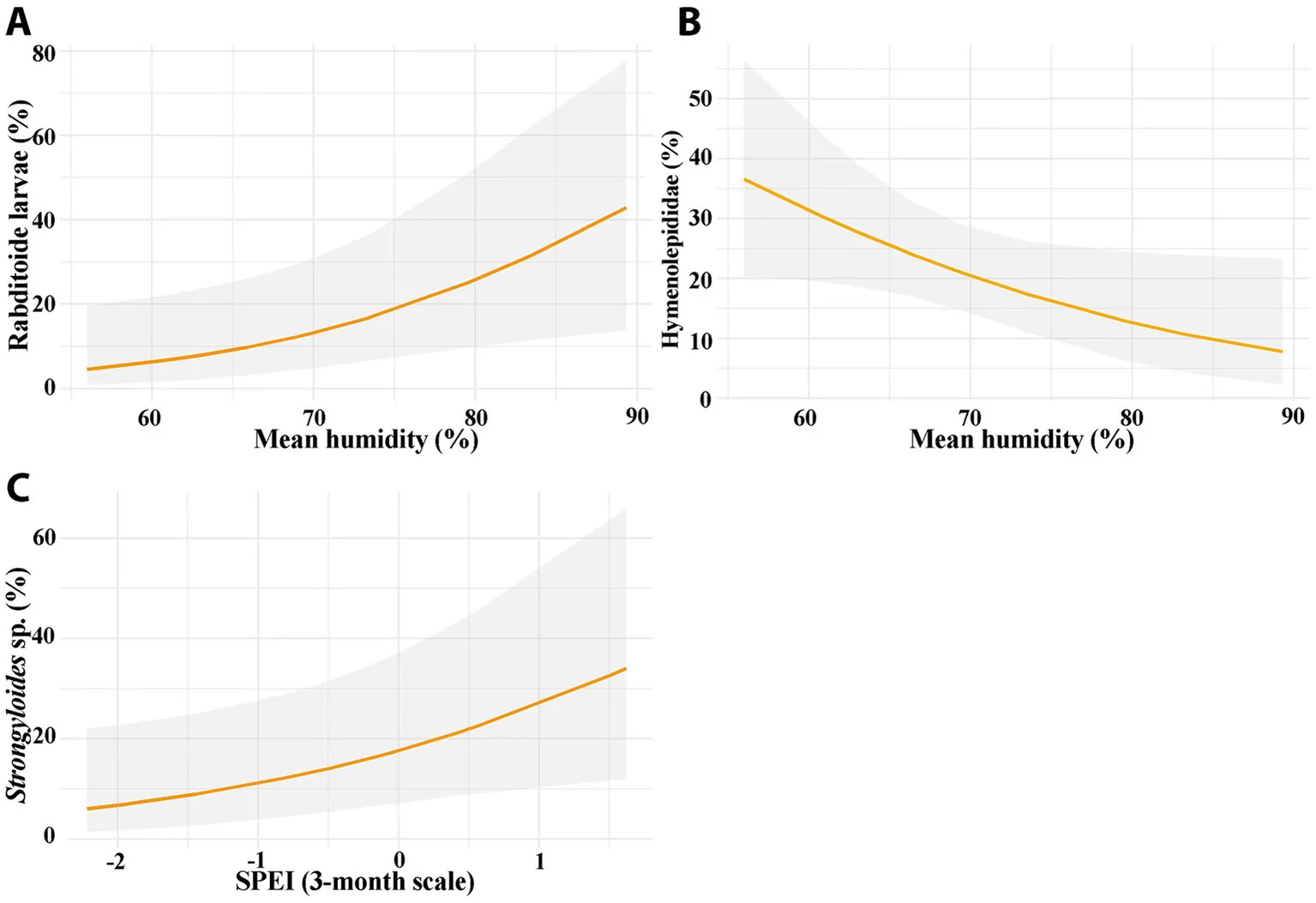
Predicted occurrence from generalized linear mixed models (GLMMs) for individual parasite taxa in *Sapajus nigritus cucullatus* from January 2019 to December 2021 in northeastern Misiones Province, Argentina: **(A)** rhabditoid larvae vs. mean relative humidity (%); **(B)** Hymenolepididae vs. mean relative humidity (%); **(C)** *Strongyloides* sp. vs. Standardized Precipitation Evapotranspiration Index (SPEI). Gray shading represents 95% confidence intervals.

Residual diagnostics indicated adequate fit for all final models, with no significant departures from uniformity, dispersion, or the absence of outliers, residual temporal autocorrelation, and no zero-inflation in the richness model (Supplementary Figures S1, S2).

## 4 Discussion

Higher SPEI values were associated with significantly higher overall gastrointestinal parasite frequency and richness in *S. n. cucullatus*. SPEI was also the main predictor of *Strongyloides* sp. occurrence. However, the influence of moisture on parasite dynamics was not captured by a single variable: mean relative humidity emerged as a significant predictor for Hymenolepididae and Rhabditoid larvae. Categorical season was not significant in any model. Taken together, these results suggest that continuous moisture-related variables (SPEI, relative humidity) outperform discrete seasonal classifications, while different parasite taxa appear to respond to different facets of the moisture regime (72, 73).

SPEI captured aspects of the moisture regime that the traditional four-season scheme largely missed, because it integrates water balance rather than treating moisture and temperature as fixed seasonal labels (27, 28). The fact that SPEI and relative humidity were only weakly correlated is biologically meaningful: relative humidity reflects the atmospheric conditions experienced instantaneously by parasite stages once shed, whereas SPEI reflects the cumulative water deficit or surplus of previous months — that is, whether the soil and leaf litter where free-living stages actually persist have been wet long enough to favor survival and infectivity (14, 74, 75). Such cumulative, lagged effects of moisture deficits on parasite transmission dynamics have been documented in other host–parasite systems using standardized drought indices at comparable multi-month temporal scales (31, 32, 76). The stronger negative correlation between SPEI and mean temperature fits this interpretation, since drought episodes in this region coincide with warmer, more evaporative periods that push moisture-sensitive transmission stages further from optimal conditions (26, 77, 78).

The positive association between SPEI and both parasite frequency and richness is consistent with literature showing that humid conditions are associated with the survival and transmission of gastrointestinal parasite infective stages (6, 12, 16, 72). By integrating precipitation, temperature, and humidity into a single measure of water balance, SPEI reduces analytical challenges associated with collinearity among these variables and also provides a biologically meaningful proxy for the moisture conditions experienced by free-living parasite stages (14, 27, 28, 79). However, the positive association between SPEI-3 and parasite richness should be interpreted with some caution. Although the relationship was statistically significant, the effect was comparatively borderline, with the confidence interval only marginally excluding the null value. Moreover, multiple parasite-community and taxon-level models were evaluated, which increases the possibility of obtaining marginally significant results by chance. Therefore, the association between SPEI-3 and parasite richness should be regarded as suggestive rather than definitive, particularly given the limited number of independent monthly climatic conditions.

Our study period (2019–2021) coincided with one of the most severe hydrological droughts recorded across South American biomes, which began as regional moisture deficit in 2019 and intensified with the onset of a multiyear La Niña event in 2020 (26, 71, 77). The 2019–2022 episode has been ranked among the top five drought events of the past seven decades in southeastern South America (26), with documented ecological impacts on adjacent freshwater and wetland ecosystems (77, 80). This natural climatic variability provided a useful gradient of moisture conditions, with SPEI values ranging from extreme drought (SPEI < −2.0) to moderately wet conditions (SPEI > + 1.5) within a single multi-annual sampling design. Because no extreme wet events (SPEI > + 2.0) occurred, the models do not allow inference about parasite dynamics under flooding conditions, which may have distinct ecological effects on parasite transmission (81).

The response observed for *Strongyloides* sp. is consistent with their direct life cycle in which free-living larvae develop in the soil before infecting the host (82). Moist soils are essential for larval survival and development, making this genus highly sensitive to environmental moisture (5, 83). This pattern aligns with previous findings in capuchin monkeys, where high *Strongyloides* sp. prevalence has been associated with humid environments (53, 84). SPEI thus appears to capture the moisture conditions associated with *Strongyloides* sp. ocurrence.

The contrasting responses of rhabditoid larvae and Hymenolepididae to relative humidity reveal ecological differences in how parasites interact with environmental conditions. Rhabditoid larvae, representing free-living stages of several nematode species, showed a strong positive association with relative humidity. This is expected, as moisture prevents desiccation and increases survival of these environmentally exposed stages (5, 83), indicating that humidity may be a key limiting factor for their persistence in the forest floor.

In contrast, the negative association between Hymenolepididae and mean humidity likely reflects an indirect ecological mechanism mediated by host feeding behavior rather than a direct effect on parasite biology (Table 1; Figure 4B). Hymenolepididae are cestodes with indirect life cycles requiring arthropod intermediate hosts (85, 86). In *S. n. cucullatus*, invertebrate consumption increases during cooler and drier periods when fruit availability declines (47, 87, 88). Agostini et al. (53) reported higher Hymenolepididae frequency in winter (35–50%) than in summer (25%) in *S. nigritus* populations in the Parana Atlantic Forest. Similar links between dietary shifts and parasite exposure have been documented in other primates, where increased consumption of invertebrates elevates the risk of acquiring trophically transmitted parasites (12, 89, 90). Lower humidity conditions may therefore be associated with increased foraging on arthropods, potentially elevating exposure to infected intermediate hosts and offering a plausible explanation for the observed pattern.

The lack of significant effects of discrete seasonal categories (summer, autumn, winter, spring) suggests that these classifications do not adequately capture the environmental variability relevant to parasite dynamics in this system. This finding supports our prediction that, in subtropical systems lacking a clearly defined dry season, continuous water-balance indices such as SPEI outperform discrete seasonal categories, in contrast to tropical systems where marked wet–dry cycles structure parasite transmission (18, 19).

The coincidence of the study period with the 2019–2021 regional drought and subsequent La Niña event provided an opportunity to examine how parasite dynamics varied under prolonged drought conditions. During extended dry periods, capuchins in the study area have been observed to modify their behavior, including the use of bamboo as alternative sources of water and food (52). Such behavioral responses to drought stress may alter host–parasite encounter rates and immune function (91–94). The lower parasite frequency and richness observed under drought conditions (negative SPEI values) may therefore reflect both reduced environmental survival of infective stages and indirect effects mediated by host behavior and physiology. As climate models project increasing frequency and intensity of extreme climatic events in South America (35), understanding how such variability influences wildlife parasitism becomes increasingly relevant. The SPEI framework offers a standardized and widely applicable tool for incorporating moisture dynamics into ecological and epidemiological analyses (27, 28).

Several limitations should be considered when interpreting these results. Because SPEI and relative humidity were derived at monthly resolution, all samples collected within a given month shared identical climatic values and consequently, although 131 fecal samples were analyzed, the environmental predictors corresponded to only 14 independent months × year combinations. In addition, samples derived from two social groups and could not be assigned to individually identified animals, so repeated sampling of the same individuals across campaigns cannot be excluded; social group and year were included as random effects to partially account for this non-independence. Although this approach captures short- to medium-term moisture variability, future studies could further refine these relationships by incorporating time-lagged analyses to evaluate delayed effects of climatic conditions on parasite dynamics. The 3-month SPEI already integrates conditions over a rolling temporal window, partially accounting for such lag effects. However, the irregular sampling intervals imposed by COVID-19 restrictions limited the temporal resolution available for formal lag analyses, which would ideally require continuous monthly sampling. Additionally, incorporating host-level variables such as body condition or physiological stress indicators (e.g., fecal glucocorticoids) could help disentangle direct environmental effects on parasite free-living stages from indirect effects mediated by host susceptibility (95, 96).

Overall, our findings suggest that wetter hydroclimatic conditions were associated with greater parasite detection, and to a lesser extent, parasite taxa richness. This distinction is important because statistical significance does not necessarily imply strong ecological effects, particularly when the number of independent climatic conditions is limited. In this context, the observed associations provide evidence that hydroclimatic variability may influence parasite dynamics, but they should not be interpreted as demonstrating a causal effect of climate on parasite transmission. By capturing continuous variation in water balance, SPEI provides a biologically meaningful proxy for the conditions experienced by parasite free-living stages and may be particularly valuable in subtropical regions, where traditional seasonal frameworks may fail to represent climatic complexity. Incorporating such indices into wildlife disease ecology can improve the ability to detect, interpret, and predict parasite dynamics under ongoing environmental change.

## Data Availability

The raw data supporting the conclusions of this article will be made available by the authors, without undue reservation.
