# Supplementary material for: Hydroclimatic variability is associated with gastrointestinal parasite dynamics in black-horned capuchins (Sapajus nigritus cucullatus) in the Atlantic Forest of Argentina
Source: Front Vet Sci. 2026 Oct 2;13:1959428. doi: 10.3389/fvets.2026.1959428 (PMC13673069; doi:10.3389/fvets.2026.1959428)
Supplement: Supplementary file 1 [file Table_1.DOCX]

Supplementary Material

**Supplementary table 1.** Number of samples collected for *Sapajus nigritus cucullatus* (n = 131) by month and year, and study site/group (COR = Corredor Biológico Urugua-í–Foerster; YL = Yacutinga Lodge).

|  | **Year** | **Month** | **COR** | **YL** | **Total** |
| --- | --- | --- | --- | --- | --- |
| **Phase I** | 2019 | May | 1 | 0 | **51** |
|  |  | Jun | 2 | 0 |  |
|  |  | Aug | 4 | 6 |  |
|  |  | Sep | 0 | 5 |  |
|  |  | Oct | 0 | 2 |  |
|  |  | Nov | 1 | 15 |  |
|  |  | Dec | 1 | 14 |  |
|  | 2020 | Jan | 4 | 16 | **43** |
|  |  | Feb | 5 | 4 |  |
|  |  | Mar | 14 | COVID-19 pandemic |  |
| **No sampling: March–December 2020 (COVID-19 pandemic)** | | | | | |
| **Phase II** | 2021 | Jan | 4 | 2 | **37** |
|  |  | Apr | 4 | 3 |  |
|  |  | Jul | 3 | 5 |  |
|  |  | Oct | 8 | 8 |  |
|  | **Total** |  | **51** | **80** | **131** |

**Supplementary figure 1.** Residual diagnostics for the generalized linear mixed models (GLMMs) of overall parasite community metrics in *Sapajus nigritus cucullatus*: parasite frequency (top) and parasite richness (bottom), both modelled as a function of SPEI-3.

**Supplementary figure 2.** Residual diagnostics for the taxon-level generalized linear mixed models (GLMMs) in *Sapajus nigritus cucullatus*: rhabditoid larvae (top) and Hymenolepididae (middle), modelled as a function of mean relative humidity, and *Strongyloides* sp. (bottom), modelled as a function of SPEI-3.
